# Porphyrins as cofactors in enzyme-catalyzed organic reactions

**DOI:** 10.52601/bpr.2025.250006

**Published:** 2025-10-31

**Authors:** Rui Wang, Jinghui Zhang

**Affiliations:** 1 Hefei National Laboratory for Physical Sciences at the Microscale and Department of Chemistry, University of Science and Technology of China, Hefei 230026, China

**Keywords:** Porphyrins, Cofactors, Artificial metalloenzymes, Enzyme-catalyzed, Organic reactions

## Abstract

Porphyrins, a class of cyclic compounds featuring a metal ion at the core of their macrocyclic structure, have long been recognized as indispensable cofactors in natural enzymatic processes. These porphyrin-based enzymes enable a wide variety of complex biochemical transformations under mild conditions with high yield, regioselectivity and stereoselectivity. As mimics of P450 enzymes, the integration of porphyrins into artificial enzymatic systems to catalyze unnatural organic reactions represents a rapidly advancing research area. Such reactions, those typically catalyzed by small molecule catalysts, are of significant interest in synthetic chemistry. This mini-review explores the role of porphyrins in both enzyme-catalyzed natural and unnatural organic reactions, with a focus on their use as cofactors in engineered metalloenzymes.

## INTRODUCTION

Porphyrins derive from the Greek word porphura, indicating the purple color in ancient times. They represent a series of macrocyclic rings composed of four pyrrole subunits linked by four methine bridging groups. The central parent structure, known as porphin, contains 18 π-electrons in its aromatic system, resulting in strong spectral absorption (Boscencu *et al.* 2023; Giovannetti 2012; Nikolaou *et al.* 2024). Owing to their excellent optical and electronic characteristics, porphyrin research has covered various fields, including P450-related biocatalysis, photodynamic therapeutic agents (Tsolekile *et al.* 2019), bioimaging probes (Ding *et al.* 2016), organic photovoltaic cells (Mahmood *et al.* 2018), nonlinear optical materials, organometallic methodologies (Barona-Castaño *et al.* 2016) and so forth.

**Figure 1:**
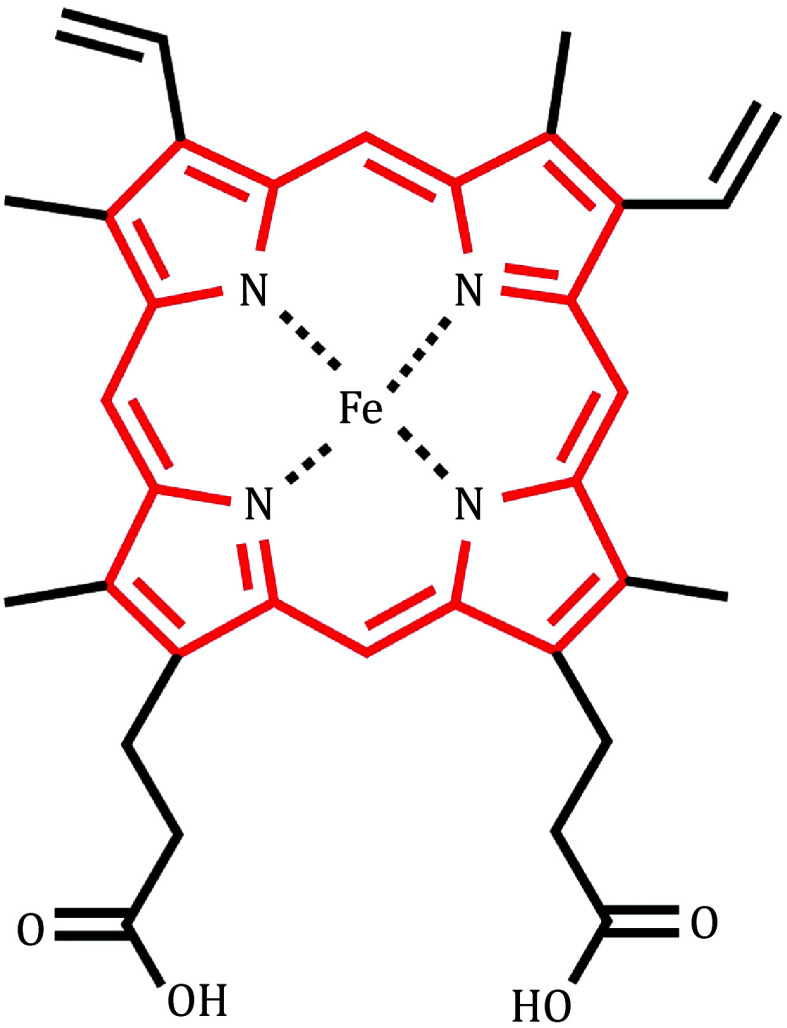
The structure of heme (iron protoporphyrin IX)

In biological systems, porphyrins serve as the cofactors including heme, vitamin B_12_, and chlorophyll in enzymes, which are responsible for oxygen transport, metabolic process and energy transformation, separately (Brothers and Senge 2022; Gruber *et al.* 2011; Lindsey 2015). Among these enzymes, heme-based P450 proteins have been extensively studied, leading to the development of a wide variety of engineered enzymes capable of catalyzing numerous reactions with excellent enantio- and diastereoselectivity. Inspired by the catalytic activity of heme protein, research groups incorporate porphyrins into different kinds of protein scaffolds to create artificial enzymes (Markel *et al.* 2021; Oohora *et al.* 2013), offering a versatile and tunable approach to biocatalyst discovery. Porphyrins, with their flexible coordination chemistry, play critical roles in the development of advanced catalytic strategies. In this mini-review, we mainly focus on the catalytic reactions functionalized by natural heme proteins like P450 enzymes and their mutants, as well as artificial enzymes containing metal porphyrins as cofactors. The structure of heme is shown in Fig. 1.

## HEME PROTEINS IN NATURE AND UNNATURAL CATALYSIS

**Figure 2:**
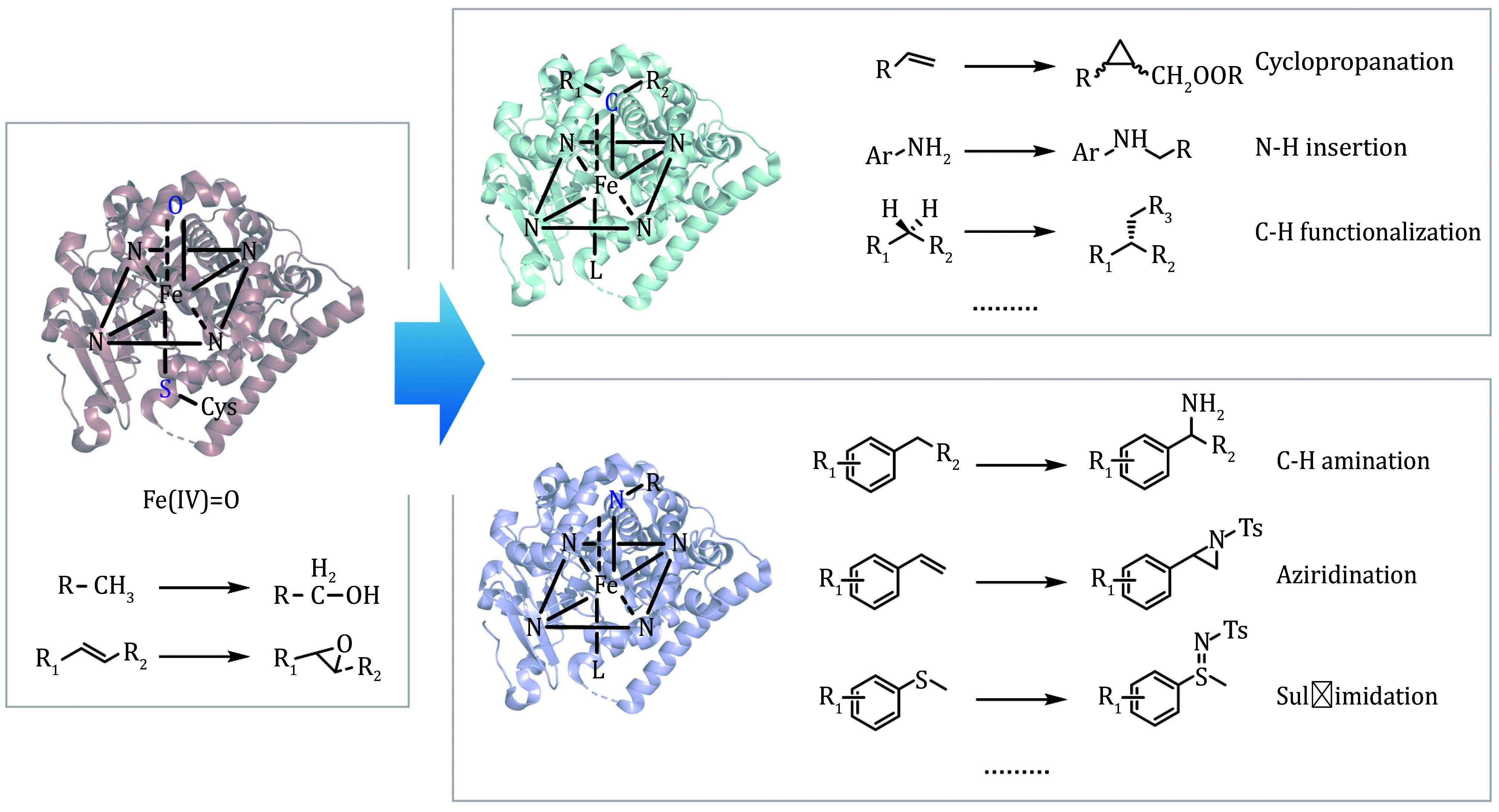
The structure of Fe oxene, carbene, nitrene and their reactions catalyzed by hemeproteins

Porphyrins have been long recognized as essential cofactors in natural enzyme catalysis. One of the best-known examples is cytochrome P450 enzymes which are a large family of oxidative heme proteins (Schuler and Sligar 2007). The term "P450" derives from the characteristic absorption peak at 450 nm observed when carbon monoxide (CO) binds to the reduced heme complex of these enzymes. P450 enzymes catalyze a range of organic transformations, including hydroxylations, epoxidations, dehydrogenations, and other oxidative processes (Podust and Sherman 2012; Schröder *et al*. 2023) with high chemo-, regio-, and stereoselectivity. They usually participate in oxygenation reactions of which the mechanism has been elucidated by the high valent iron-oxo intermediate species (Denisov *et al*. 2005; Groves *et al*. 1979; Guengerich and Macdonald 1990). A number of scientists including the Arnold group have made great efforts to investigate and explore the reactions from Fe oxene intermediates Fe(IV)=O and subsequently introduced Fe carbenes and Fe nitrenes intermediates similar to Fe(IV)=O. The formation of metal-carbenoid and metal-nitrenoid intermediates from azide and diazo precursors enables P450 enzymes to catalyze reactions without the need for external reductants. As a result, various types of reactions for carbene and nitrene transfer have been demonstrated with the wild and engineered heme proteins, largely expanding the repertoire of hemin enzymes. The structure of Fe Oxene, Carbene, and Nitrene are shown in Fig. 2.

In particular, the Arnold group produced the variants of the cytochrome P450 from *Bacillus megaterium* (CYP102A1, or P450_BM3_) as the efficient catalysts for the asymmetric metallocarbene-mediated cyclopropanation of styrenes (Coelho *et al*. 2013). This work gave the inspiration that highly active variants for specific substrate can be generated via a wealth of beneficial directed mutation. Further screening variants enable P450s to achieve N-H insertion by metal carbenoids (Wang *et al*. 2014), C-H functionalization (Jia *et al*. 2020) and other unnatural reactions.

It can be observed that by substituting other amino acid residues for axial ligation to the iron center, the axial mutants exhibit different spectral features and electronic properties. The cytochrome P411s, another de-variant of cytochrome P450_BM3_ was first created by the Arnold group (Coelho *et al*. 2013)*,* featuring a serine axial ligand to the heme iron in place of cysteine ligand from the wild type. The mutants are successful in carry out cyclopropanation with extremely high conversion in the whole system, providing an easier route for the evolution of enzyme biocatalysts. Surprisingly, in the following studies, the P411s show their great potential for a series of new-to-nature reactions that P450s have no access to, especially for the carbene and nitrene transfer reactions (Liu *et al*. 2021; Mao *et al*. 2024; Yang *et al*. 2019). Apart from the P450s enzyme, cytochrome *c* from *Rhodothermus marinus* is also investigated to exhibit high specificity for carbon-silicon bond formation with excellent stereoselectivities (Kan *et al*. 2016). Notably, Fasan’s group is also fascinated by heme proteins and demonstrated many successful designs and catalytic activities of the heme-based catalysts (Singh *et al*. 2015; Steck *et al*. 2020). For example, they conducted a panel of evolutionary screenings of myoglobin to achieve the gram-scale synthesis of tasimelteon and a TRPV1 inhibitor (Bajaj *et al*. 2016), demonstrating the potential for practical applications of heme enzymatic catalysis.

The above highlights several examples of heme enzymes and their fabulous catalytic activity and selectivity towards natural and unnatural reactions. These remarkable advances provoke the great interest of scientists to discover a wider variety of desirably selective products through the directed evolution of heme enzymes.

## ARTIFICIAL METALLOENZYMES IN BIOMIMETIC CATALYSIS

Artificial metalloenzymes (ArMs), as complementary to natural enzymes, have emerged as a new approach to discovering more catalytic activities, making remarkable progress in recent years (Lewis 2013; Schwizer *et al*. 2018). By incorporating metal catalysts into protein scaffolds, artificial metalloenzymes have the potential to endow chemical reactions with favorable selectivity specificity. The catalytic activity of a metalloenzyme is determined by both the primary coordination sphere of the metal and the protein scaffold as the secondary coordination sphere. This large hydrophobic substrate binding pocket around the metal is where the reactive site is located, finely tuning the selectivity of the reaction by surrounding amino acid residue. Numerous artificial enzymes have been developed as excellent catalysts for new-to-nature reactions, where porphyrin-based ones attracted much attention owing to their biomimetic catalysis ability. However, when heme is used as the catalytic center in enzymes, the enzymes need to be precisely provided to ensure that the heme can be stably embedded. The pocket must not only accommodate the spatial structure of the heme but also provide suitable amino acid residues to stabilize the iron center, enabling effective interaction with the substrate. Therefore, finding or designing an appropriate hydrophobic pocket is one of the key challenges in assembling porphyrin-containing artificial enzymes. Through techniques such as protein engineering, computational modeling, and high-throughput screening, scientists can design and optimize the hydrophobic pocket in a more effective way.

**Figure 3:**
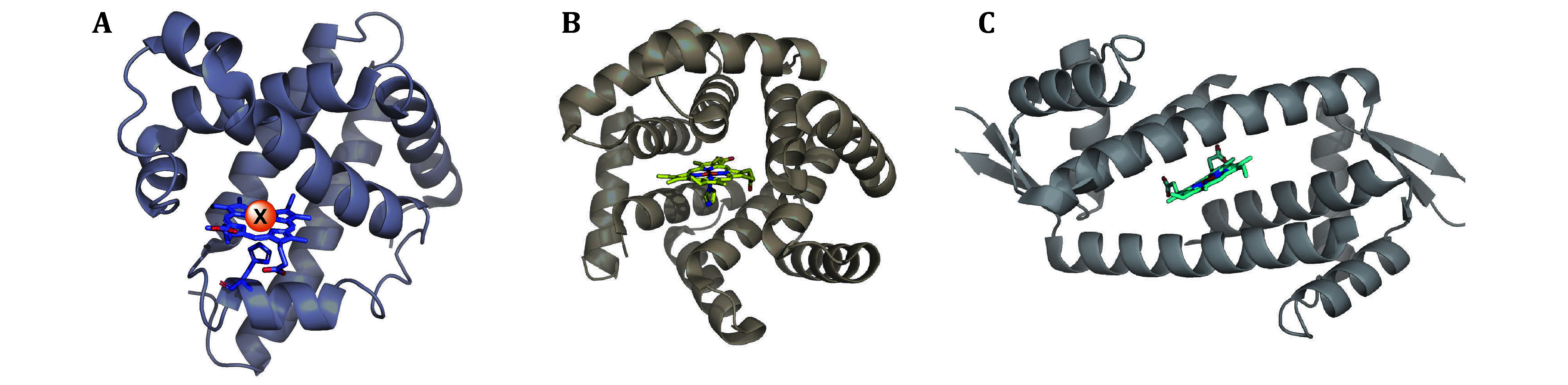
**A** Image of P450 enzyme (PDB ID: 1MBN). **B** The crystal structure of dnHEM1 (PDB ID: 8C3W). **C** The crystal structure of LmrR⊂heme (PDB ID: 6FUU)

Hartwig group developed a new strategy by combining the abiological metal with a natural heme protein scaffold. To do so, they first created a series of metal–porphyrin cofactors containing Co, Cu, Mn, Rh, Ir, Ru, and Ag *in vitro*, and then incorporated them into the apo-form of myoglobin to generate various artificial enzymes (Key *et al*. 2016). Among them, Ir(Me)-myoglobin and its mutants show high reactivity towards C-H activation (Dydio *et al*. 2016; Gu *et al*. 2019) and cyclopropanation with varying diastereoselectivity and enantioselectivity. The new strategy of metal substitution of iron coordinated with the porphyrin cofactors has emerged as a promising tool to expand the reaction space. Metal substitution can offer more opportunities for designing porphyrin-containing artificial enzymes, while the choice of open substrate binding pocket is still limited. Gerard Roelfes’s group, however, employed a lactococcal multidrug resistance regulator (LmrR) as the scaffold to be self-assembled with heme. The resulting LmrR⊂heme complex is capable of catalyzing abiological enantioselective cyclopropanation reactions (Villarino *et al*. 2018). Though the pocket of LmrR is fully occupied by the binding of the cofactor heme, the dynamic space between heme and tryptophan in LmrR enabled the artificial enzyme good activity and enantioselectivity in catalysis. This work suggests the key to creating an efficient artificial enzyme is the absence of a defined binding site for the substrate in the dynamic space between the pocket and the cofactor. In view of this, Indrek Kalvet and co-workers utilized the helical bundle scaffolds (Regan and DeGrado 1988) with a large tunable central cavity which possesses good substrate tolerance, and incorporated heme into them. The de novo protein, dnHEM1, is elaborately designed with the help of computational modeling and calculation, generating enantio-complementary carbene transferases for styrene cyclopropanation (Kalvet *et al*. 2023). The images of three artificial enzymes are shown in Fig. 3.

The incorporation of porphyrins into a protein scaffold can enhance its ability to bind substrates with high specificity, mimicking the selectivity of natural enzymes. This versatility enables the development of highly selective catalysts that can perform synthetic transformations under mild conditions, offering a greener alternative to traditional chemical processes.

## CONCLUSION

In summary, with the involvement of numerous enzyme types and a wide range of reactions catalyzed by porphyrin-containing enzymes, porphyrins as cofactors demonstrate tremendous potential in enzymatic catalysis. Their ability to facilitate a broad range of complex organic transformations, including stereoselective C−C, C−N, C−B, and C−Si bond-forming processes, makes them capable of reactions that small-molecule catalysts cannot achieve. Considering the large number of enzymes in the P450 family, the structural diversity of porphyrins (including axial variability), and the vast enzyme pockets yet to be explored, numerous opportunities and possibilities for porphyrin-containing biocatalysts in unnatural reactions remain to be uncovered. Despite the many remarkable achievements of wild-type and artificial porphyrin enzymes, several challenges remain. Firstly, it seems to be ambiguous to predict the direction of the evolution, and several generations of mutants need to be tested before finding an optimal mutation. Secondly, the choice of precise protein scaffolds for specific substrates and products is difficult, which may be time-consuming and require tremendous trial and error. To address these issues, much more attention should be focused on the integration training of experimental data and advanced computational methods like machine learning algorithms. Moreover, state-of-the-art techniques like high-throughput screening platforms should be finely employed to streamline the screening process. The exploration of these areas holds great promise for advancing the field of biocatalysis and developing innovative solutions for various chemical and biological applications.

## Conflict of interest

Rui Wang and Jinghui Zhang declare that they have no conflict of interest.
